# Polarization-Enhanced Underwater Laser Range-Gated Imaging for Subaquatic Applications

**DOI:** 10.3390/s24206681

**Published:** 2024-10-17

**Authors:** Shuaibao Chen, Peng Liu, Wei He, Dong Luo, Yuguang Tan, Liangpei Chen, Jue Wang, Qi Zhao, Guohua Jiao, Wei Chen

**Affiliations:** 1 College of Engineering, Southern University of Science and Technology, 1088 Xueyuan Avenue, Shenzhen 518055, China; sb.chen@siat.ac.cn; 2 Shenzhen Institute of Advanced Technology, Chinese Academy of Sciences, Shenzhen 518055, China; 3 University of Chinese Academy of Sciences, Beijing 100049, China

**Keywords:** range-gated image, polarized light, image enhancement, underwater application

## Abstract

Laser range-gated underwater imaging technology, by removing most of the backscattering noise, can effectively increase image contrast and extend the detection range. The optical signal captured by a range-gated imaging system primarily comprises reflected light from the object and backscattered light from the surrounding water. Consequently, surfaces with low reflectivity or highly turbid water environments substantially constrain the applicability of the range-gated imaging system. To enhance the detection capability of underwater laser range-gated imaging, this paper proposes the incorporation of underwater polarized light imaging technology as an enhancement method. Based on polarization differences, backscattered light and reflected light from an object can be distinguished. Experimental results indicate that, compared to images obtained using a conventional range-gated laser imaging system, those captured with a polarization-enhanced system exhibit an increase of up to 47% for the corresponding Enhancement Measure Evaluation (EME) index. The proposed approach, which integrates polarization imaging with range-gated laser imaging, has the potential to broaden the applicability of underwater laser imaging scenarios, such as deep-sea exploration and military applications.

## 1. Introduction

In ocean exploration, the acquisition of image data has become an essential component for comprehensive regional exploration and for resource extraction activities, such as mining and oil extraction [1]. Underwater imaging research primarily encompasses two main branches: optical imaging and acoustic imaging. Compared to acoustic imaging, optical imaging provides superior resolution and contrast.

Due to the complex underwater environment, light experiences significant absorption, scattering and turbulence during propagation [2]. This explains why conventional underwater optical images exhibit significantly lower contrast, increased blurring and greater distortion compared to images captured in air. Therefore, there is substantial potential for advancement in the restoration and enhancement of underwater images. To mitigate noise in underwater images, researchers have investigated optimization techniques including polarization imaging [3], binocular imaging [4] and LiDAR imaging [5].

In 1995, researchers first trained octopuses to distinguish between targets based on the presence or absence of a pattern created by a 90° polarization contrast within the target [6]. Based on the experimental results, it was found that polarized vision could provide information analogous to color vision, thereby enhancing the detection and recognition of objects. Subsequently, Yoav Y. Schechner and Nir Karpel developed a model of light transmission in water by adapting an existing model for air, and proposed a computer vision method that integrates the target image captured by using a polarizer [7].

In 2008, Tali delineated the role of active light sources in polarization imaging [8]. Since then, more specialized imaging methods have been proposed, building upon previous models. To achieve optimal image pairs, some researchers have utilized the maximum peak difference in the optical correlation plane within reconstruction programs [9,10]. Researchers estimate background light using various methods, including parameter relationships across estimating the polarized-difference image [11], polynomial fitting [12], Gaussian surface fitting combined with least squares [13] and hierarchical image analysis integrated with wavelet variations [14]. Based on the properties of underwater polarized light, methods such as traversal optimization for Degree of Polarization (DOP) values [15], periodic polarized image integration [16] and the application of Gaussian low-pass filters [17] or Butterworth filters [18] have been reported to enhance restoration algorithms. The laser range-gated imaging technique was first introduced to underwater imaging in 1967 [19], when Georges’ team developed the initial corresponding system [20]. Over the years, the technology has undergone significant updates and iterations, including the development of three-dimensional range-gated imaging [21] and the introduction of more compact and practical systems, such as UTOFIA [22] and LUCIE3 [23]. In recent years, polarization imaging has become an important area of research for image enhancement in underwater imaging. Polarization imaging demonstrates significant advantages in reducing scattering, enhancing image contrast and recovering details. Particularly in situations where background areas or prior information are not available [24], it can effectively improve the image recovery quality of moving targets by utilizing the intensity and degree of the polarization of global or local backscattered light. Some studies have also introduced rapid processing techniques, such as optical correlation methods and image downsampling, which significantly increase computational speed [25]. Additionally, in air, gated imaging technology has been combined with methods to shield non-target scattered light, along with deep prior and denoising algorithms, to enhance imaging under foggy conditions [26]. Gated imaging can effectively remove backscattering through the principle of spatial slicing; however, it requires significant power from active light sources. In contrast, polarization imaging can reduce some background scattered light but also depends on the characteristics of the target object.

To address the limitations associated with laser range-gated imaging, this study investigates the integration of range-gated technology with polarization imaging techniques. The objective is to enhance the image clarity of the target. By optimizing the range-gated polarization system, this approach aims to enable its deployment in diverse aquatic environments and adapt its use across various applications, including resource exploration.

## 2. Materials and Methods

### 2.1. Principles of Polarization-Enhanced Range-Gated Imaging Technology

#### 2.1.1. Polarization Enhancement

The optical propagation model in aqueous media is fundamentally analogous to that in gaseous media. The camera primarily captures two components of illumination: ambient light and light reflected from the target. A simple monochrome model for light transmission in water is

(1) $$I\left(x,y\right)=D\left(x,y\right)+B\left(x,y\right)=T\left(x,y\right)t\left(x,y\right)+B_{\infty }\left(x,y\right)\left(1-t\left(x,y\right)\right),$$

where *I* denotes the light intensity incident upon the receiving end, *D* represents the light reflected by the object and *B* signifies the background illumination arriving at the receiving end. *T*, *t* and $B_{\infty }$ represent the light distribution of the object, the transmittance of the water medium and the backscattered light from the infinitely distant water, respectively. Regarding polarization, there are various representations, including the Jones matrix, Mueller matrix and Stokes vector, among others. In this study, Stokes parameters are utilized to describe the polarization state of the light field, enabling the separation of reflected light from the object and backscattered light from the water.

(2) $$S=\left[\begin{array}{c} I\\ Q\\ U\\ V \end{array}\right]=\left[\begin{array}{c} I_{0^{{}^{\circ}}}+I_{90^{{}^{\circ}}}\\ I_{0^{{}^{\circ}}}-I_{90^{{}^{\circ}}}\\ I_{45^{{}^{\circ}}}-I_{135^{{}^{\circ}}}\\ I_{\mathrm{left}\;}-I_{\mathrm{right}\;} \end{array}\right].$$

In Equation (2), *I* represents the total irradiance, independent of the polarization state; *Q* denotes the difference between the time-averaged irradiances measured with a linear polarizer oriented at 0° and 90°; *U* signifies the difference between the time-averaged irradiances measured with a linear polarizer oriented at 45° and 135°; and *V* is defined as the difference between the time-averaged irradiances of right and left circularly polarized light. The Stokes vector provides a quantitative representation of the polarization state of light.

Building on this expression, researchers have introduced a parameter to characterize the polarization state of light, known as the DOP, which is computed as follows:

(3) $$DOP=\frac{I_{\max}-I_{\min}}{I_{\max}+I_{\min}}.$$

Consequently, the DOP for the reflected polarized light from the target object and the DOP for the background polarized light from the water can be determined as specified in Equations (4) and (5), respectively.

(4) $$DOP_{target}=\frac{D_{\max}-D_{\min}}{D_{\max}+D_{\min}},$$

(5) $$DOP_{scatter}=\frac{B_{\max}-B_{\min}}{B_{\max}+B_{\min}}.$$

In the Stokes matrix formalism, ΔI can be derived as follows:

(6) $$\Delta I=I_{\max}-I_{\min}.$$

Based on Equation (1), the expression for *D* can be reformulated as follows:

(7) $$T=\frac{I-B_{\infty }}{t}+B_{\infty }.$$

Considering both Equations (4) and (5), $B_{\infty }$ can be expressed as

(8) $$B_{\infty }=\frac{\Delta I-D\cdot DOP_{target}}{DOP_{scatter}\cdot \left(1-t\right)}.$$

Finally, integrating all the aforementioned equations, the reflected light from the target object can be expressed as follows:

(9) $$T=\frac{I\cdot DOP_{scatter}-\Delta I}{t\cdot (DOP_{scatter}-DOP_{target})}.$$

#### 2.1.2. Range-Gated Imaging

Underwater optical imaging without active illumination is significantly influenced by the aquatic environment, which often fails to meet the requirements for effectively imaging underwater targets in diverse aquatic conditions. However, with the use of active illumination, the light reflected from the target and received by the camera often contains significant amounts of backscattered light generated by the water body. Therefore, the elimination of backscattered light is the focus in the study of underwater optical imaging. Taking into account the time difference between the arrival of backscattered light from the water and the reflected light from the target object at the receiver, researchers have explored a range-gated imaging method to mitigate the background scattered light. The fundamental principle involves configuring the gate at the receiving end to open at a specific time, thereby permitting only the light within the depth of field where the reflected light from the target object is located to be detected.

According to [27], the model for the energy detected by the receiver in a laser range-gated system can be expressed as follows:

(10) $$E_{1}=K\cdot \frac{B_{r}}{r^{2}}\cdot E_{0}exp\left(-2\sigma r\right),\left.r\in \left[\begin{array}{c} R_{1},R_{2} \end{array}\right.\right],$$

where *K* denotes the proportional impact factor associated with the light’s path, $B_{r}$ represents the sensitive area of the camera, and *r* represents the distance between the camera and the target, ranging from $R_{1}$ to $R_{2}$, within which the reflected light can be detected by the system. Based on the research of underwater light propagation [28], by integrating a polarizer in the optical path, the backscattered light accumulated at the receiving end, situated at a distance *r* from the range-gated system, can be expressed as follows:

(11) $$I_{b}=\int_{R_{1}}^{R_{2}}E\cos\left(\theta \right)dr,$$

where θ represents the angle of the polarizer.

### 2.2. Principle Verification Experiment

#### 2.2.1. Image Evaluation Indicators

Since relying on a single-image evaluation metric often leads to inaccurate assessments, multiple-image evaluation metrics were employed in the experiment. To quantitatively assess the image quality, we analyzed the image contrast. According to [7], the expression for contrast is given by

(12) $$C\left(I\right)=\sum_{\delta }\delta {\left(x,y\right)}^{2}P_{\delta }\left(x,y\right),$$

in which δ(x,y) represents the gray-level difference between adjacent pixels, while $P_{\delta }\left(x,y\right)$ denotes the probability distribution function of the gray-level differences between adjacent pixels. Contrast measurement serves as a fundamental parameter for determining the modulation transfer function of the medium and has been demonstrated to correlate with underwater visual performance.

Another metric employed for image evaluation is information entropy, which quantifies the average amount of information contained within the image. And according to [29], it can be calculated as

(13) $$H\left(I\right)=-\sum_{i}\sum_{j}p_{i,j}\log p_{i,j}.$$

In the equation, $p_{i,j}$ represents the proportion of the gray value of the pixel within the image. Additionally, as described in the literature [30], a quantitative method is introduced to assess the enhancement effect of an image. This method is used to select the optimal parameters and transformations for each enhancement technique, with the Enhancement Measure Evaluation (EME) calculated as follows:

(14) $$EME\left(T\right)=\frac{1}{kl}\sum_{k_{1}=0}^{k}\sum_{k_{2}=0}^{l}20log\frac{T_{max;k_{1},k_{2}}}{T_{min;k_{1},k_{2}}+q},$$

where *k* and *l* denote the number of segments in the horizontal and vertical directions of the image, respectively. $T_{max}$ and $T_{min}$ represent the maximum and minimum gray values within each image segment, while *q* is a correction factor that prevents the occurrence of a minimum gray value of 0, which could otherwise lead to a failure in the EME calculation. The main role of the EME metric is to evaluate image contrast and enhancement effects, particularly when assessing the effectiveness of image enhancement algorithms for low-light images. It provides quantitative information on contrast improvement.

#### 2.2.2. Practical Parameter Acquisition in Polarization-Enhanced Range-Gated Imaging Experiment

From the preceding section, to obtain a clear target image requires determining the mathematical expressions for the optical transmission matrix *t*, the degree of polarization of the reflected light from the target, and the degree of polarization of the reflected light from the surrounding water environment. At the first step, histogram equalization is applied to the two input images, designated as $I_{max}$ and $I_{min}$. Based on the assumption that high-frequency information primarily represents the reflected light from the target, and low-frequency information primarily represents the reflected light from the water environment in the polarization-selective image, the high-frequency and low-frequency components of $I_{max}$ and $I_{min}$ are separated by using an ideal filter. This could be achieved through the application of an ideal high-pass filter and an ideal low-pass filter, as described below:

(15) $$H_{0}\left(\mu \right)=\left\{\begin{array}{cc} 1, & \mathrm{if}\;|\mu |\leq D_{0}\\ 0, & \mathrm{if}\;|\mu |>D_{0} \end{array}\right.,$$

(16) $$H_{1}\left(\mu \right)=\left\{\begin{array}{cc} 1, & \mathrm{if}\;|\mu |\geq D_{0}\\ 0, & \mathrm{if}\;|\mu |<D_{0} \end{array}\right..$$

In the equation, $H_{0}\left(\mu \right)$ and $H_{1}\left(\mu \right)$ represent the filter responses in the frequency domain, where μ is the frequency variable and $D_{0}$ denotes the cutoff frequency. In the experiment, μ was set to 1. Convolution was then performed between the input image and the filter frequency response, as described by the following:

(17) $$I_{fre}=F^{-1}\left\{F\left\{I\right\}\times H\left(u,v\right)\right\}.$$

The high-frequency and low-frequency information of the image obtained could be used to determine the value of the DOP. On one hand, the separated high-frequency image was used to calculate $DOP_{target}$, representing the degree of polarization of the target’s reflected light. Additionally, a parameter factor $\epsilon_{0}$ was introduced to adjust the calculation result to more closely approximate the true value. On the other hand, the separated low-frequency image was used to calculate $DOP_{scatter}$, which represents the degree of polarization of the background light. This value was adjusted to reflect the actual conditions through polynomial fitting and an additional factor $\epsilon_{1}$. The expression for the background light grayscale value polynomial fitting is as follows:

(18) $$I\left(x,y\right)=ax^{2}+by^{2}+cxy+dx+ey+f.$$

In the equation, a,b,c,d,e and *f* all represent the coefficients to be determined. Considering the limited spatial extent of the selected region within the water body, we assumed that the optical properties of the water in this small area are uniform, and that the value of *t* is consistent within a plane perpendicular to the direction of transmission. Thus, the corrected expression for the reflected light from the target is given by

(19) $$D=\frac{I\cdot \epsilon_{1}DOP_{scatter}-\Delta I}{t\cdot (\epsilon_{1}DOP_{scatter}-\epsilon_{0}DOP_{target})}.$$

Since both the values of DOP and *t* range from 0 to 1, an evaluation index score can be introduced by partitioning the parameter interval and conducting optimization calculations. This approach helps to identify the influence factor value and the corresponding *t* value that maximize the evaluation index for the enhanced image. The expression for the evaluation index is as follows:

(20) $$\begin{array}{cc} score(\epsilon_{0},\epsilon_{1},t) & =a\times Normalized_{EME}+b\times Normalized_{entropy}\\ \; & +c\times Normalized_{contrast}. \end{array}$$

In the equation, a,b and *c* are undetermined coefficients, which could be set to 0.4, 0.3 and 0.3, respectively, during the construction of the target board image. Furthermore, $Normalized_{EME}$, $Normalized_{Entropy}$ and $Normalized_{Contrast}$ represent the normalized values obtained through the following equations:

(21) $$\begin{array}{cc} \; & Normalized_{EME}=\frac{EME-EME_{min}}{EME_{\max}-EME_{\min}},\\ \; & Normalized_{Contrast}=\frac{Contrast-Contrast_{min}}{Contrast_{\max}-Contrast_{\min}},\\ \; & Normalized_{Entropy}=\frac{Entropy-Entropy_{min}}{Entropy_{\max}-Entropy_{\min}}. \end{array}$$

The optimal values of $\epsilon_{0}$, $\epsilon_{1}$ and *t* can be determined through the following:

(22) $$\epsilon_{opt0},\epsilon_{opt1},t=\arg\max\left\{score\left(\epsilon_{0},\epsilon_{1},t\right)\right\}.$$

**Figure 1:**
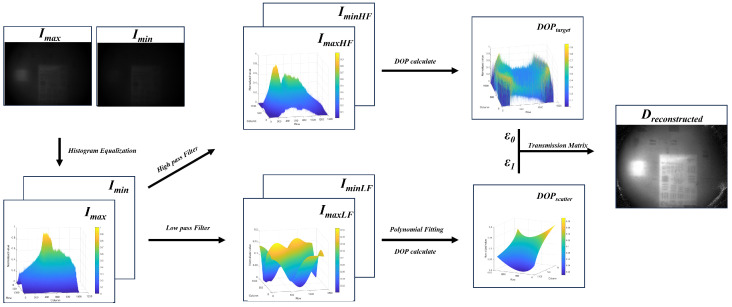
Program workflow of the polarization enhancement algorithm.

Figure 1 illustrates the overall practical procedure, using mutually orthogonal target polarization images as an example.

**Figure 2:**
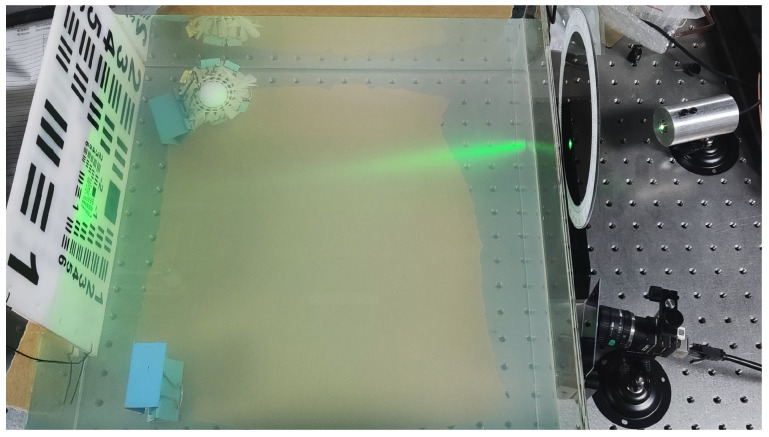
Polarization camera used to validate the polarization enhancement algorithm.

To verify the image enhancement algorithm, a pre-experiment using a polarized camera was conducted. Initially, we conducted a functional verification of the polarization image enhancement algorithm. The input images consisted of $0^{{}^{\circ}}$ and $90^{{}^{\circ}}$ polarization images acquired by a Lucid camera (PHX050S-QC), which captures images with different polarization states using a polarization mask at the pixel level. The target was a resolution test target made of acrylic. The imaging environment consisted of an acrylic water tank containing water with dimensions of 11.2 cm × 39.0 cm × 39.0 cm. The experimental setup is shown in Figure 2. To simulate water environments with different turbidity levels in actual scenarios, we added a certain amount of milk to the water tank to validate the effectiveness of image enhancement techniques on images captured under these conditions.

**Figure 3:**
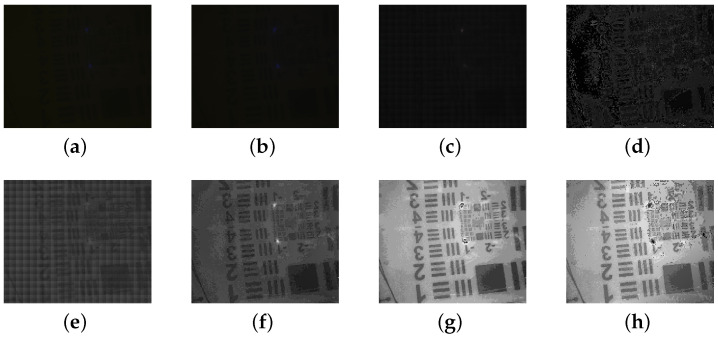
Original image and algorithm-enhanced image. (**a**) Normal polarized image of $0^{{}^{\circ}}$. (**b**) Normal polarized image of $90^{{}^{\circ}}$. (**c**) Original image. (**d**) Reconstruced image by Schechner’s method $90^{{}^{\circ}}$. (**e**) Reconstruced image by CLAHE. (**f**) Reconstruced image by polynomial fitting. (**g**) Reconstruced image by Butterworth filter. (**h**) Reconstruced image by method in the paper.

The target images were enhanced using various methods, as illustrated in Figure 3, based on input images with mutually orthogonal polarization. These methods include Schechner’s approach [7], traditional CLAHE [31], polynomial fitting [12], Butterworth low-pass filtering [18] and the techniques described in this article.

As illustrated in Figure 3, the target object is nearly invisible in both conventional camera images and polarized images. For polarized images, the image enhancement method presented in this article distinctly reveals the fine details of the stripes. Comparing the image’s yellow enhancement results obtained from various methods, it is evident that the enhancement method proposed in this study reveals more pronounced detailed features compared to Schechner’s method, and its results are generally consistent with those obtained using the Butterworth filter.

By calculating picture evaluation metrics such as EME, contrast and entropy for each image shown in Figure 3, we obtain the results presented in Table 1.

Based on this table, it is evident that the EME of the enhanced images obtained using this method shows a significant improvement compared to the EME values of images enhanced using other polarization enhancement methods. The contrast and entropy values demonstrate clear advantages over both the original image and the results from other enhancement methods. Overall, the results demonstrate that the polarization image enhancement method proposed in this article is effective and beneficial for enhancing underwater images.

#### 2.2.3. Verification of the Polarization State Received in the Range-Gated System

**Figure 4:**
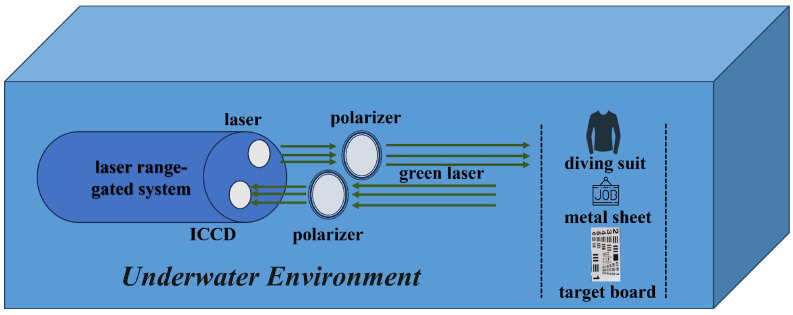
Scene of the image taken by a polarization camera.

The laser range-gated imaging experiment, which utilized polarized light enhancement, was conducted in a pool with an approximate depth of 1 meter. The experimental setup is shown in Figure 4. The range-gated system emits a 532nm illumination laser which passes through the water and a polarizer successively to ensure that the illumination light has good linear polarization state. Laser light transmits through the water body and is then reflected by the target. The reflected light passes through another polarizer before reaching the gated camera. The angle of a polarizer that the reflected light passes through can be rotated from $0^{{}^{\circ}}$ to $360^{{}^{\circ}}$.

**Figure 5:**
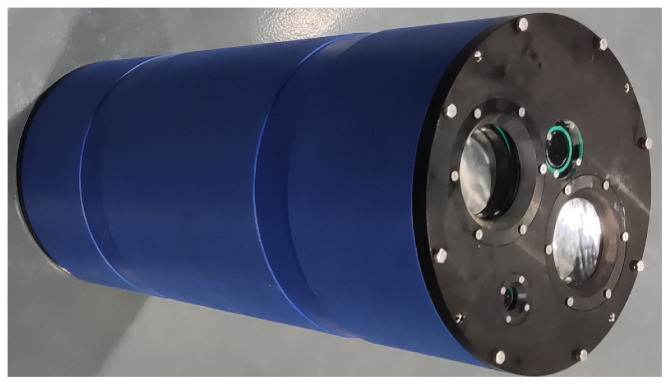
Range-gated system.

**Figure 6:**
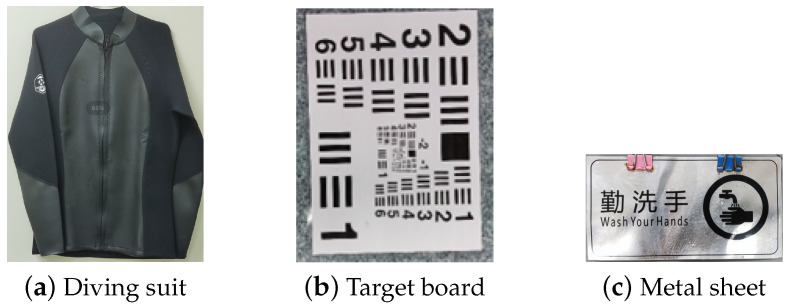
Experiment imaging target.

The range-gated imaging system is shown in Figure 5. It emits pulsed laser with a repetition frequency of 2 kHz and a pulse width of 1.5 ns. The outer side of the polarizer has a scale that allows for the precise display of the polarization angle. The polarization-enhanced range-gated imaging experiments were conducted in a 9.1 m×1.7 m×1.0 m pool. The three targets employed in the experiment are a diving suit, a target board and a metal sheet, as depicted in Figure 6.

The entire photo capture experiment can be divided into two phases. In the first phase, a green laser with a wavelength of 532 nm was utilized, and the target object was positioned 5 meters from the range-gated imaging system. Subsequently, 100 mL and 200 mL of milk were added to the water, respectively. We then captured images of the object at polarizer’s angles of $0^{{}^{\circ}}$, $45^{{}^{\circ}}$, $00^{{}^{\circ}}$ and $135^{{}^{\circ}}$ under varying turbidity conditions. The acquired images were subsequently enhanced and optimized through post-processing.

**Figure 7:**
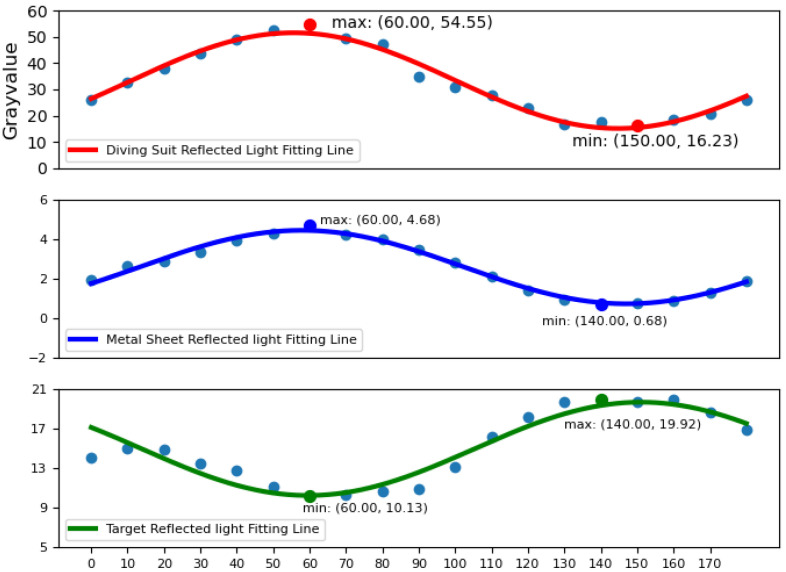
Examination of the preservation of the polarization state of the image by adjusting the angle of the polarizer.

The optimized target image can be derived by inputting orthogonal images into the polarization enhancement program. In this experiment, images of the target were also acquired at various polarization angles ranging from $0^{{}^{\circ}}$ to $180^{{}^{\circ}}$, with increments of $10^{{}^{\circ}}$. For the three different target objects, we rotated the polarizer to collect the polarized pictures obtained from different polarization angles, and then calculated the grayscale value of each picture to obtain the fitting curves as shown in Figure 7. As the rotation angle of the polarizer changes, the fitted curves exhibit sinusoidal characteristics, indicating that the reflected light captured by the selective imaging system possesses polarization properties.

#### 2.2.4. Validation Experiment in a Small Water Tank

**Figure 8:**
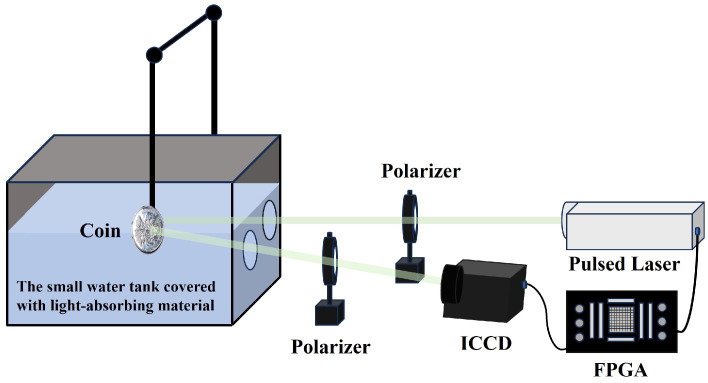
Experimental setup for polarization-enhanced laser range-gated imaging.

The enhancement effect of polarization on range-gated imaging was first tested in a small water tank measuring 60.0 cm × 12.5 cm × 28.5 cm (length × width × height), as shown in Figure 8.

**Figure 9:**
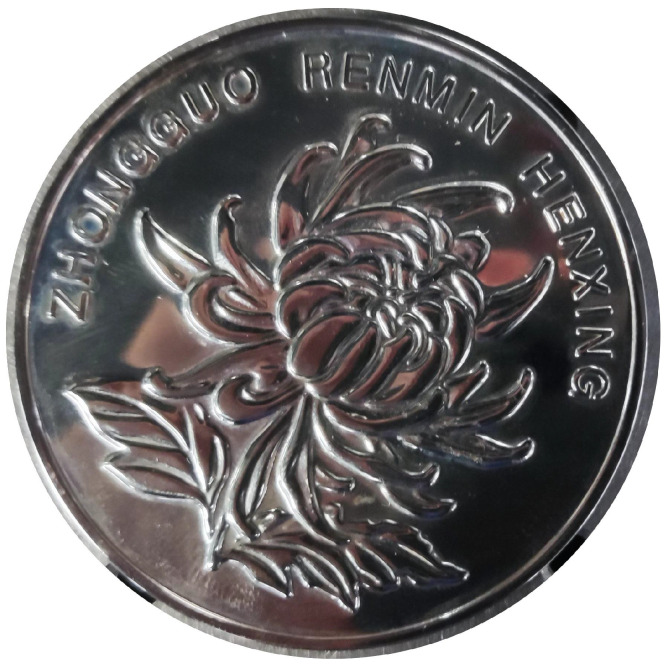
The coin used in the experiment.

The pulsed laser with a wavelength of 532 nm passed through a polarizer before entering the water tank to ensure the polarization state of the active illumination light. A rotatable polarizer is placed in front of the ICCD used for imaging to capture reflected light with different polarization states. As shown in Figure 9, the target object chosen for the experiment is a coin with patterns and text, which is placed 30 cm away from the laser incidence window. The small water tank originally contained pure water, which was then mixed with milk to alter the turbidity. For each turbidity level, the polarizer in front of the ICCD was rotated to $0^{{}^{\circ}}$, $45^{{}^{\circ}}$, $90^{{}^{\circ}}$ and $135^{{}^{\circ}}$ to capture images of different polarization states. The imaging hardware system, consisting of a pulsed laser, ICCD and FPGA, operates in a gated mode.

The verification experiment results in the small water tank are shown in Table 2 and Table 3. Table 2 displays the coin images captured using conventional range-gated imaging under different turbidity conditions, while the photos obtained with the polarization-enhanced range-gated imaging system under the same water conditions are shown below. The image comparison shows that the images captured by the polarization-enhanced system have more details and less white noise. Table 3 provides the image quality metrics for the images shown in Table 2. The results indicate that, in most metrics, the polarization-enhanced images perform better.

## 3. Polarization-Enhanced Range-Gated Imaging Experiment Results

### 3.1. Polarization Enhancement Effect under Different Material Conditions

**Figure 10:**
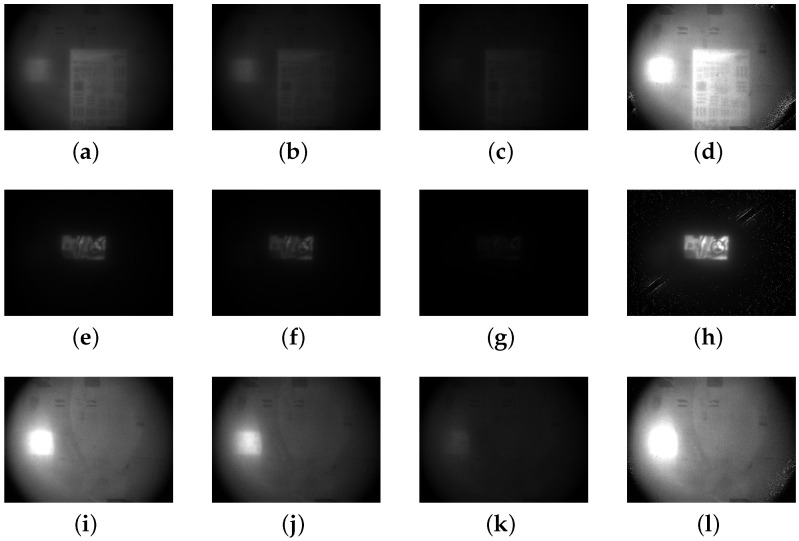
Original images of the three materials, along with images obtained and enhanced using the polarization algorithm. (**a**) Range-gated image of target board. (**b**) $I_{max}$. (**c**) $I_{min}$. (**d**) Polarization-enhanced range-gated images of target board. (**e**) Range-gated image of metal sheet. (**f**) $I_{max}$. (**g**) $I_{min}$. (**h**) Polarization-enhanced range-gated images of metal sheet. (**i**) Range-gated image of diving suit. (**j**) $I_{max}$. (**k**) $I_{min}$. (**l**) Polarization-enhanced range-gated images of diving suit.

In the second phase of the experiment mentioned in Figure 4, photographs of objects located 2.26 meters away were captured in an underwater environment with a turbidity of approximately 2.67 NTU. By rotating the polarizer, we can acquire images of the objects—including metal sheets, target boards and diving suits—at polarization angles of $0^{{}^{\circ}}$, $45^{{}^{\circ}}$, $90^{{}^{\circ}}$ and $135^{{}^{\circ}}$. For the range-gated imaging system, after acquiring polarization images of three targets at different polarization angles, and inputting the $0^{{}^{\circ}}$ and $90^{{}^{\circ}}$ images, we obtained the enhanced images of the three types of objects, as illustrated in Figure 10.

Compared to the range-gated images, the image enhancement results obtained with the polarizer generally exhibit greater clarity. From the image pairs, it is evident that the enhanced pictures reveal greater detail, including the stripes, numbers on the standard target, the swan logo on the metal sheet and the collar silhouette of the diving suit. It can be observed that the image enhancement of the metal sheet is primarily evident in the delineation of the twisted metal edges rather than in the flat, overall image shape. By calculating picture evaluation metrics such as EME, contrast and entropy for each image shown in Figure 10, we derive the results presented in Table 4.

Among the different objects used in the experiment, the polarization algorithm enhancement results for the three targets show improvements in the three indicators: EME, contrast, and entropy. Considering EME, the corresponding values for the target board image, metal sheet image and diving suit image increased by 47.47%, 32.09% and 15.63%, respectively. Additionally, with respect to the entropy index, the corresponding values for the target board image, metal sheet image and diving suit image increased by 22.26%, 37.52% and 2.36%, respectively.

Considering the enhanced images, the image enhancement effect for the target board and metal sheet images is significantly superior to that for the diving suit image, as evidenced by the EME and entropy indexes. The values of the first two are markedly higher than that of the third. It can be observed that, although both the target board and the diving suit are composed of polymeric materials, the enhancement effects differ due to the variations in the types of materials used. While the primary components of the diving suit are neoprene and nylon, the target board is made of acrylic.

### 3.2. Polarization Enhancement Effect under Different Turbidity Conditions

**Figure 11:**
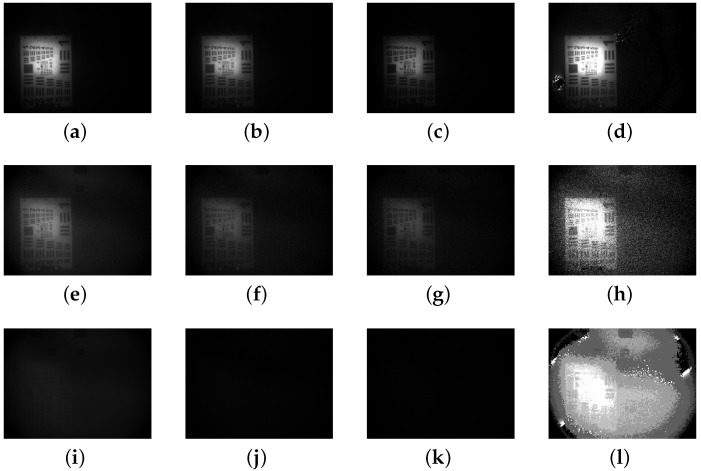
Original images of the target at different turbidities, along with images obtained and enhanced using the polarization algorithm. (**a**) Range-gated image. (**b**) $I_{max}$. (**c**) $I_{min}$. (**d**) Polarization-enhanced range-gated images of the target board under turibidity of 1.70 NTU. (**e**) Range-gated image. (**f**) $I_{max}$. (**g**) $I_{min}$. (**h**) Polarization-enhanced range-gated images of the target board under turibidity of 3.89 NTU. (**i**) Range-gated image. (**j**) $I_{max}$. (**k**) $I_{min}$. (**l**) Polarization-enhanced range-gated images of the target board under turibidity of 5.41 NTU.

As discussed in the preceding section, the target is positioned at a constant distance from the range-gated imaging system, and polarized images are acquired at varying angles through the rotation of the polarizer. The varying levels of turbidity in the environment are achieved by incorporating different quantities of milk. By inputting mutually orthogonal image pairs acquired under different turbidity conditions, the corresponding polarization-enhanced images, as illustrated in Figure 11, can be obtained.

The images below demonstrate that range-gated images, when subjected to a polarization enhancement process, exhibit improved clarity under various turbidity conditions. As the turbidity of the underwater environment increases, the enhancement effect becomes more pronounced. For instance, utilizing the polarization enhancement algorithm allows for the visibility of the target board concealed within the obscured range-gated image.

Table 5 presents the evaluation metrics of the system for images captured without polarizers as well as for enhanced images obtained through polarization enhancement. It is evident from the table that after polarization enhancement, the Enhancement Measure Evaluation (EME), contrast of the enhanced image and entropy value have shown substantial improvement.

Regarding the entropy index, the values for the target board image, metal sheet image and diving suit image increased by 6.77%, 15.58% and 37.53%, respectively. This indicates that the enhancement effect of the polarization enhancement algorithm becomes more pronounced as the turbidity of the underwater environment increases.

## 4. Conclusions

This study investigates the feasibility of integrating polarization imaging with laser range-gated imaging to optimize underwater imaging quality. It employs a comprehensive methodology, supported by specific experiments to demonstrate the efficacy of polarization imaging enhancement in enhancing the quality of range-gated imaging.

Through polarization-gated imaging experiments with various materials, it has been verified that incorporating polarization enhancement imaging yields higher-quality underwater images compared to gated imaging alone. However, the effectiveness of the enhancement varies depending on the material composition of the objects. In comparison to targets composed of plastic materials, polarization imaging has a more pronounced impact on optimizing the imaging quality of targets made from metal materials.

The experimental results of vibration image enhancement demonstrate that, compared to a standalone underwater laser range-gated imaging system, the system incorporating polarized light enhancement significantly improves the contrast and entropy values of images captured in turbid water. As the turbidity of the water body increases, the enhancement in image metrics becomes more pronounced. Experimental results indicate that as the turbidity of the water body increases, the entropy index of the same target image rises by approximately 6% to 38%. Among images of target objects composed of different materials, the enhancement in Enhancement Measure Evaluation (EME) indicators ranges from 16% to 47%.

As an effective underwater imaging technique, the range-gated imaging method combined with the polarization enhancement algorithm proposed in this study warrants further exploration and potential application in the underwater domain.

The system applies a pulsed laser with an average power of 2 W and a single-pulse energy of 2 mJ. According to UTOFIA’s report on behavioral studies on laser light source [32] on their website, this laser has no significant effect on the behavior of fish and shrimp at a distance of 1 meter, nor does it have a strong impact on fish eyes at 1 meter. Considering the environmental impact of laser usage, in the future, our system will be structurally adjusted to position the laser focus within the system itself, thereby reducing the potential impact of the laser on the environment.

Currently, this technology is limited by the acquisition of polarization images. In the future, the introduction of unsupervised learning and the combination of efficient computing power may promote its real-time application.

## Figures and Tables

**Table 1:** Evaluation metrics of images processed by different image enhancement methods.

Enhancement Method	EME	Contrast	Entropy
Original image	6.745	2.334	2.934
Schechner’s method	20.078	49.416	5.609
CLAHE	9.030	55.521	5.147
Polynomial fitting	1.590	14.651	5.618
Butterworth filter	1.509	36.140	4.713
Method in the paper	3.855	115.405	6.829

**Table 2:** Pictures from conventional range-gated imaging and polarization-enhanced range-gated imaging.

Turbidity/NTU	41.8	52	61.8	69.3
**Pictures from conventional range-gated imaging**	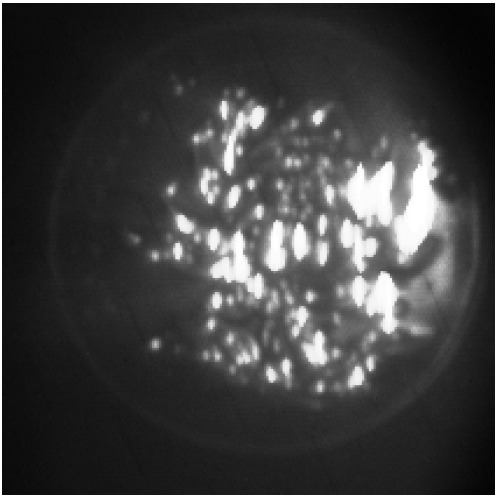	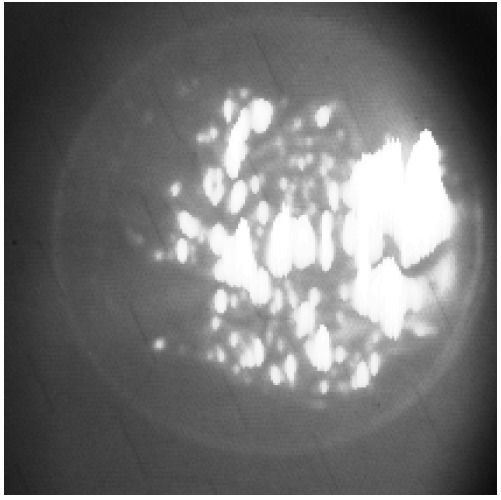	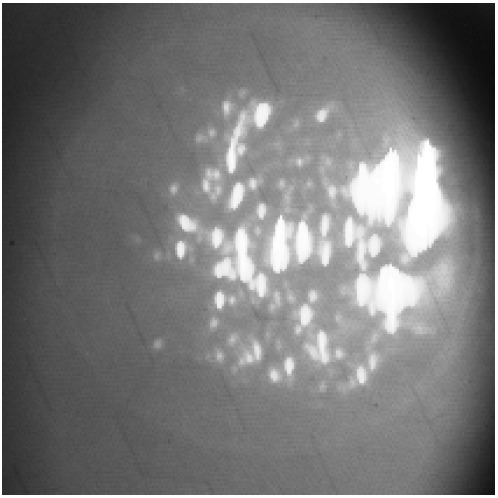	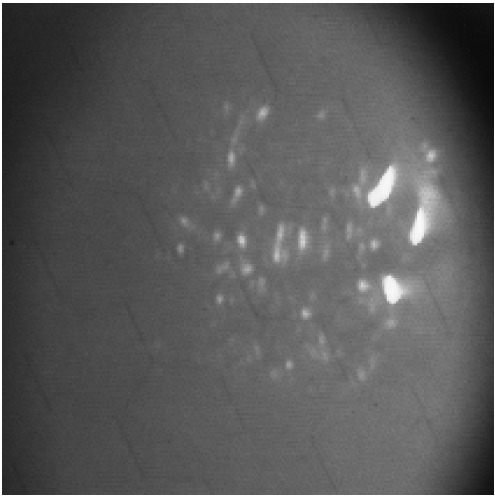
**Pictures from polarization-enhanced range-gated imaging**	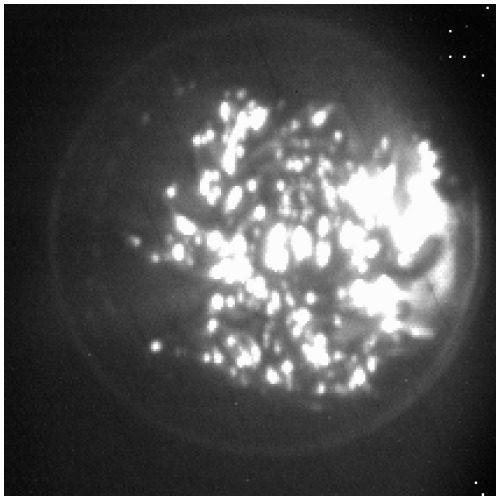	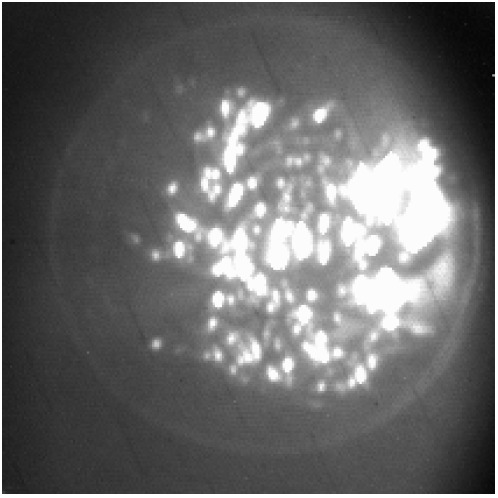	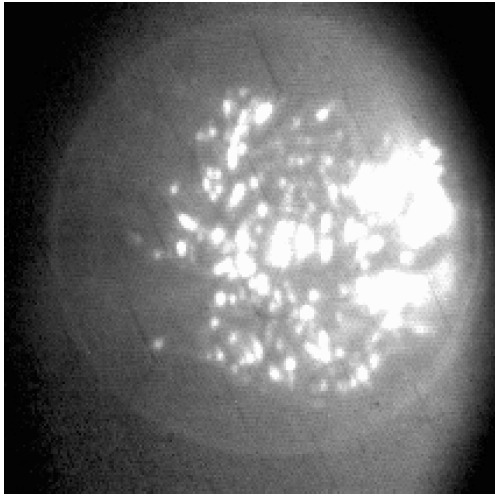	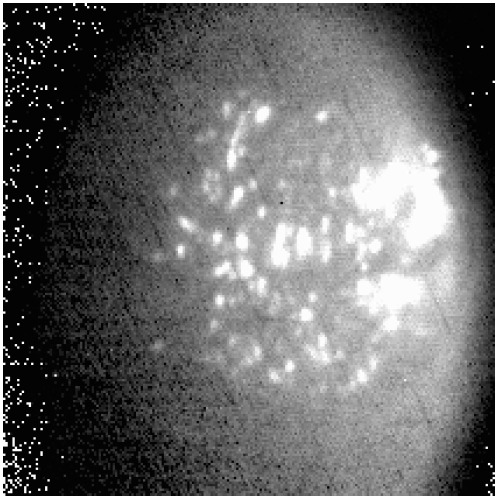

**Table 3:** Comparison of image evaluation metrics between conventional range-gated imaging and polarization-enhanced range-gated imaging.

Polarization Imaging Optimization	Turbidity (NTU)	EME	Contrast	Entropy
NO	41.8	10.575	143.894	6.985
YES	41.8	11.758	157.358	7.180
NO	52.0	5.861	75.569	7.498
YES	52.0	7.276	90.700	7.396
NO	61.8	5.073	59.639	7.492
YES	61.8	24.898	107.390	7.547
NO	69.3	4.533	27.877	6.750
YES	69.3	42.124	125.698	7.335

**Table 4:** Impact on image evaluation metrics for different targets after polarization enhancement.

Target	Polarization Enhancement	EME	Contrast	Entropy
target board	NO	10.919	4.959	6.365
	YES	16.077	310.995	7.782
metal sheet	NO	62.194	1.297	3.825
	YES	82.792	402.347	5.260
diving suit	NO	8.251	22.171	7.260
	YES	9.541	124.775	7.431

**Table 5:** Impact of polarization enhancement on image evaluation metrics under different turbidity conditions.

Polarization Enhancement	Turbidity/NTU	EME	Contrast	Entropy
NO	1.70	8.026	5.978	6.084
YES	1.70	135.374	571.302	7.032
NO	3.89	6.913	3.250	4.771
YES	3.89	28.690	28.901	5.094
NO	5.41	1.517	0.172	4.511
YES	5.41	4.421	140.437	6.204

## Data Availability

Data are contained within the article.
